# BAFF Inhibition Effectively Suppresses the Development of Anti-HLA.A2 Antibody in the Highly Sensitized Mouse Model

**DOI:** 10.3390/ijms22020861

**Published:** 2021-01-16

**Authors:** Ji Won Min, Yoo-Jin Shin, Hyeyoung Lee, Bo-Mi Kim, Ki Hyun Park, Kyoung Chan Doh, Tae-Min Kim, Sun Woo Lim, Chul Woo Yang, Eun-Jee Oh, Byung Ha Chung

**Affiliations:** 1 Division of Nephrology, Department of Internal Medicine, Bucheon St. Mary’s Hospital, College of Medicine, The Catholic University of Korea, Seoul 06591, Korea; blueberi12@gmail.com; 2 Convergent Research Consortium for Immunologic Disease, Seoul St. Mary’s Hospital, College of Medicine, The Catholic University of Korea, Seoul 06591, Korea; dbwls612@gmail.com (Y.-J.S.); kimbbomi1987@gmail.com (B.-M.K.); kchan87@nate.com (K.C.D.); swlim@catholic.ac.kr (S.W.L.); yangch@catholic.ac.kr (C.W.Y.); 3 Transplant Research Center, The Catholic University of Korea, Seoul 06591, Korea; 4 Department of Laboratory Medicine, Catholic Kwandong University International St. Mary’s Hospital, Incheon 22711, Korea; shomermaid@catholic.ac.kr; 5 Department of Molecular & Cell Biology, Graduate School, The Catholic University of Korea, Seoul 06591, Korea; neojkl1004@naver.com; 6 Department of Medical Informatics, The Catholic University of Korea, Seoul 06591, Korea; tmkim@catholic.ac.kr; 7 Division of Nephrology, Department of Internal Medicine, Seoul St. Mary’s Hospital, College of Medicine, The Catholic University of Korea, Seoul 06591, Korea; 8 Department of Laboratory Medicine, Seoul St. Mary’s Hospital, College of Medicine, The Catholic University of Korea, Seoul 06591, Korea

**Keywords:** HLA.A2 transgenic mice, sensitization, skin allograft, BAFF, donor-specific antibody

## Abstract

B cell activating factor (BAFF) is a cytokine that plays a role in the survival, proliferation and differentiation of B cells. We proposed to observe the effects of BAFF inhibition on the humoral immune responses of an allosensitized mouse model using HLA.A2 transgenic mice. Wild-type C57BL/6 mice were sensitized with skin allografts from C57BL/6-Tg (HLA-A2.1)1Enge/J mice and were treated with anti-BAFF monoclonal antibody (mAb) (named Sandy-2) or control IgG1 antibody. HLA.A2-specific IgG was reduced in BAFF-inhibited mice compared to the control group (Δ-13.62 vs. Δ27.07, *p* < 0.05). BAFF inhibition also resulted in increased pre-pro and immature B cell proportions and decreased mature B cells in the bone marrow (*p* < 0.05 vs. control). In the spleen, an increase in transitional B cells was observed with a significant decrease in marginal and follicular B cells (*p* < 0.05 vs. control). There was no significant difference in the proportions of long-lived plasma and memory B cells. Microarray analysis showed that 19 gene probes were significantly up- (>2-fold, *p* < 0.05) or down-regulated (≤2-fold, *p* < 0.05) in the BAFF-inhibited group. BAFF inhibition successfully reduced alloimmune responses through the reduction in alloantibody production and suppression of B cell differentiation and maturation. Our data suggest that BAFF suppression may serve as a useful target in desensitization therapy.

## 1. Introduction

Sensitization to human leukocyte antibody (HLA) is an important obstacle to overcome for favorable long-term post-transplant allograft survival. According to the US data, up to 35% of patients on the waiting list for a transplant are sensitized [1]. This situation is similar in Korea, hence 15.4% of patients on a waiting list for kidney transplant showed high sensitization to HLA in terms of positive crossmatch [2]. Percentage graft loss in sensitized patients has been shown to be much worse compared to compatible patients [3]. Several desensitization protocols, such as the use of plasmapheresis, rituximab, intravenous immune globulin (IVIg), and bortezomib, are being used and are under trial in these patients [4]. However, donor-specific antibody (DSA) generation and antibody-mediated allograft injury still remains an unresolved problem awaiting better therapies [5].

Meanwhile, B cell activating factor (BAFF) is a cytokine that plays a role in the survival, proliferation and maturation of B cells [6,7]. Previous studies have found that elevated BAFF levels are significantly associated with disease activity of various types of immune disorders such as chronic graft versus host disease [8], systemic lupus erythematosus (SLE), Sjögren disease, multiple sclerosis and rheumatoid arthritis [9,10]. BAFF levels have also been reported to significantly predict post-transplant clinical outcomes [11,12]. For example, pre-transplant soluble BAFF levels showed correlation with the de novo appearance of DSA [13], or with increased incidence of antibody-mediated rejection (ABMR) and lower rejection free allograft survival [14]. In our previous report, we found that pre- and post-transplant serum BAFF level showed significant association with sensitization to HLA [15]. All of the above data strongly suggest that therapy targeting BAFF may help prevent sensitization to HLA. Indeed, monoclonal antibodies binding BAFF (i.e., Tabalimumab and Belimumab) are currently in use or under trial for use in several autoimmune diseases [9,10] and have been tried for use in desensitization therapy for kidney transplant [16] as well as for use in the maintenance of immunosuppression [17].

Based on this background, we investigated whether inhibition of BAFF can prevent the development of anti-HLA antibody using a well-established sensitized mouse model to HLA-A2 [18,19]. For this, we measured anti-HLA-A2 antibody titers using the luminex single antigen assay in mice with or without BAFF inhibition, and also analyzed the phenotype of B cell lineage in the spleen and bone marrow to observe the changing pattern of immune cells according to anti-BAFF treatment. Lastly, we investigated molecular signatures using microarray to observe the changes in transcripts associated with the development or suppression of sensitization to HLA.

## 2. Results

### 2.1. Comparison of Specific IgG Responses to Skin Allograft in Each Group

**Figure 1:**
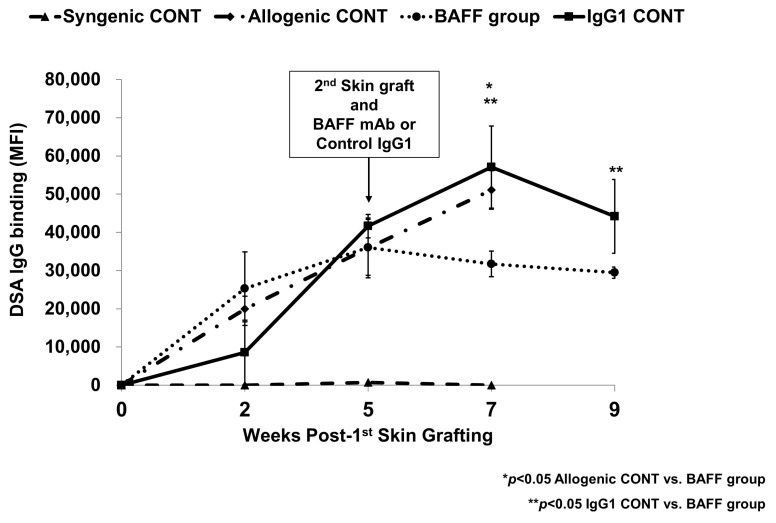
Mean fluorescence intensity (MFI) titers of HLA.A2-specific IgG measured at week 2, 5, 7 and 9. Error bars represent 2 standard errors (SE).

A sensitized mouse model was used to observe the effects of BAFF inhibition in allo-sensitization. Briefly, two skin grafts from a C57BL/6-Tg(HLA-A2.1)Enge/J mouse were transplanted to a wild-type C57BL/6 mouse. BAFF inhibition was achieved by injecting a BAFF monoclonal antibody (mAb) in the second allogenic TP and BAFF inhibitor group (BAFF group) just before the second skin transplantation. The difference in the single transgenic HLA.A2 antigen was expected to evoke detectable allogenic immune responses in the recipient animals. DSA responses were observed with serum samples at weeks 0 (before first skin graft), 2, 5 (before second skin graft), 7 (2 weeks after second graft), and 9 (4 weeks after second graft) in the skin graft recipients. As shown in Figure 1, mean fluorescence intensity (MFI) titers of anti-HLA A2 Ab was hardly detected in the second syngenic TP (Syngenic CONT) group (Week 5, 720 ± 1239; Week 7, 9.2 ± 6.7) while in the allogenic transplant groups, MFI titers reached 30~40 thousand by the fifth week just before the second skin transplant. After administration of BAFF mAb, titers in the BAFF group decreased (Δ − 13.62) in the seventh week but kept rising in the second allogenic TP (Allogenic CONT) (Δ29.64) (*p* < 0.05 vs. BAFF group) and second allogenic TP and control IgG1 (IgG1 CONT) (Δ27.07) (*p* < 0.05 vs. BAFF group). The difference in MFI titers persisted until week 9 where PRA levels were still significantly higher in the IgG1 CONT group compared to the BAFF group (MFI titer IgG1 CONT 36,086 vs. BAFF group 29,479, *p* < 0.05). Therefore, we observed that BAFF inhibition successfully reduced humoral responses in terms of anti-HLA-A2 Ab.

### 2.2. Comparison of B Cell Fractions in the Bone Marrow

**Figure 2:**
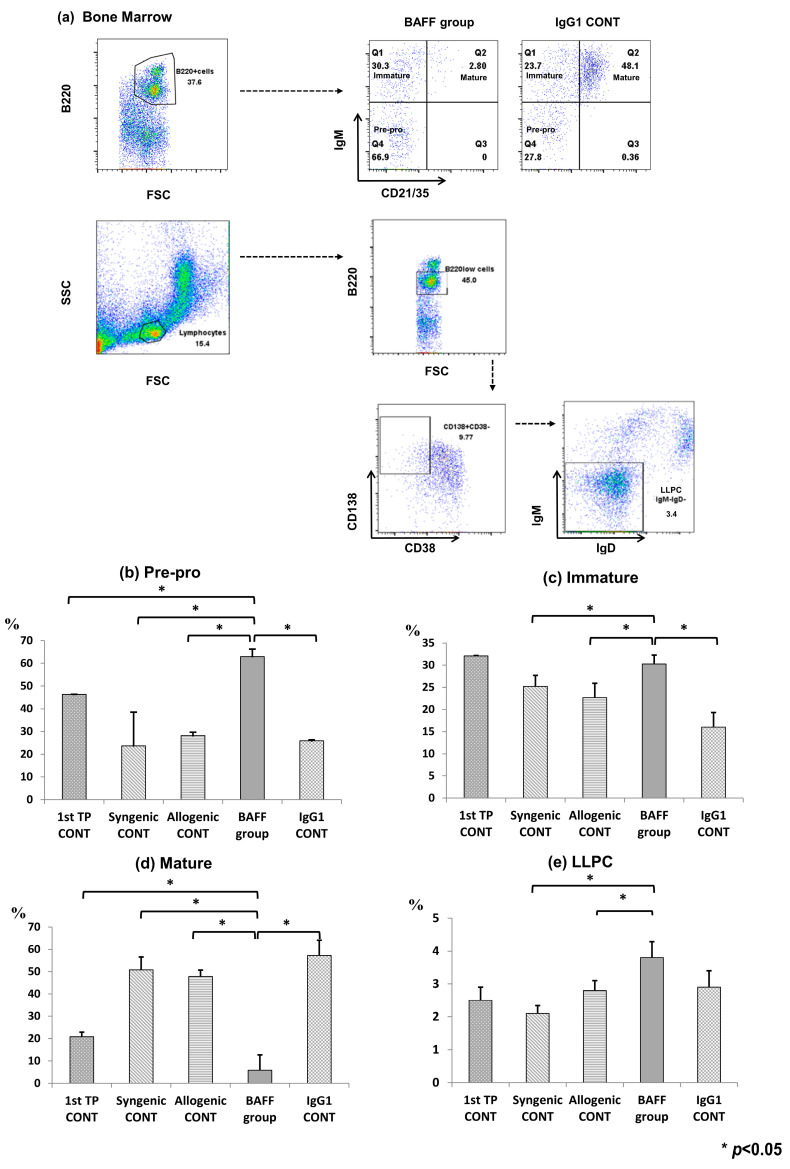
B cell population at week 9 (4 weeks after second transplantation) in the recipient bone marrow analyzed using flow cytometry. (**a**) Gating strategy, (**b**) fractions of B220+CD21/CD35-IgM-pre-pro B cells, (**c**) B220 + CD21/CD35-IgM+immature B cells, (**d**) B220 + CD21/CD35 + IgM+ mature B cells, and (**e**) B220lowCD138 + CD38low(Ig–) long lived plasma cells (LLPC). Error bars represent 2 standard errors (SE).

To substantiate the effects of BAFF inhibition on activation and maturation of B cells in the bone marrow, fractions of B cell subsets in all five groups were analyzed using flow cytometry (Figure 2a–e). Immature cell fractions (Pre-pro and Immature B cells) were significantly increased in the BAFF group compared to the other groups (Pre-pro: BAFF group 63.0 ± 5.4 vs. IgG1 CONT 25.9 ± 2.1, *p* < 0.05, Figure 2b) (Immature: BAFF group 30.3 ± 2.4 vs. IgG1 CONT 16.0 ± 3.3, *p* < 0.05, Figure 2c). In contrast, the proportion of mature cells was significantly suppressed in the BAFF group compared to the control groups (BAFF group, 5.9 ± 2.4 vs. IgG1 CONT 57.2 ± 5.2, *p* < 0.05, Figure 2d). The proportion of long-lived plasma cells (LLPC) was obviously lowest in the unsensitized, Syngenic CONT group and was increased in the BAFF group compared to the allogenic CONT group (first allogenic TP (1st TP CONT) 2.8 ± 0.7 vs. Syngenic CONT 2.1 ± 0.4 vs. Allogenic CONT 2.6 ± 0.5 vs. BAFF group 3.8 ± 1.4, *p* < 0.05; BAFF group vs. IgG1 CONT 2.9 ± 0.9, *p* = 0.052, Figure 2e).

### 2.3. Comparison of B Cell Fractions in the Spleen

**Figure 3:**
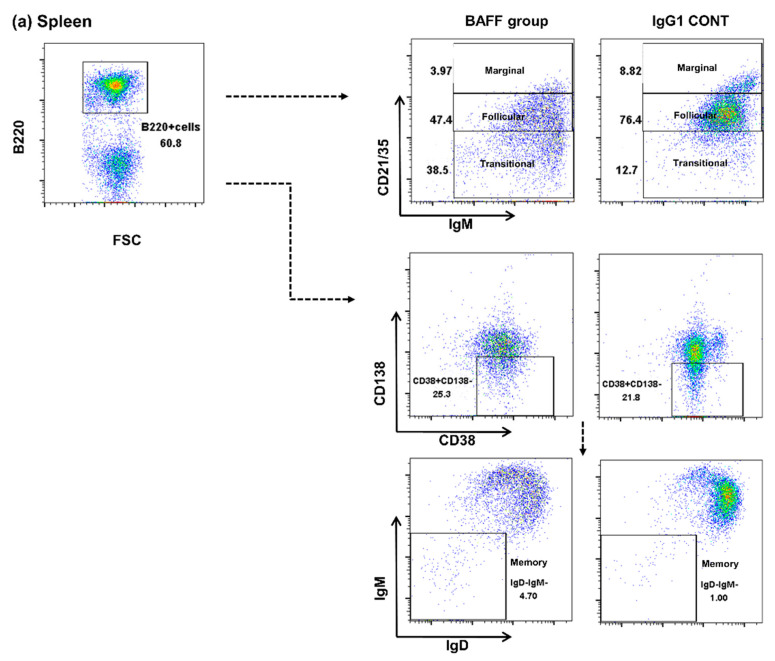

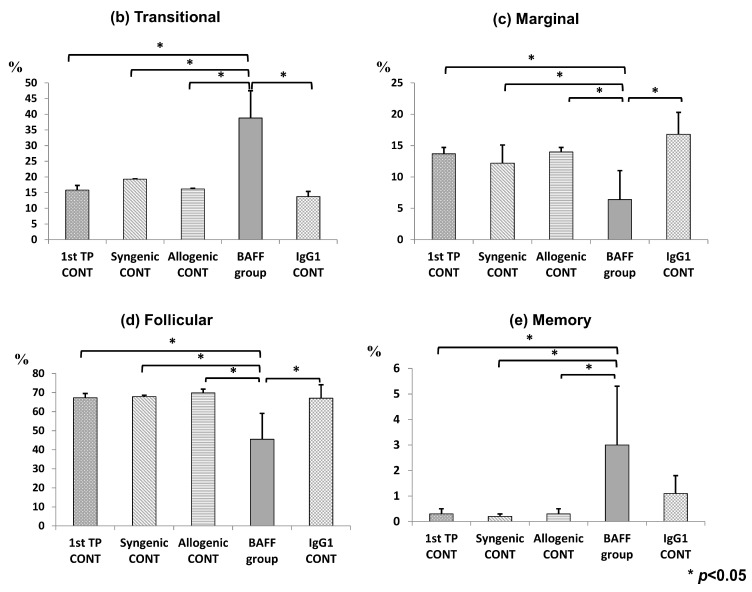
B cell population at week 9 (4 weeks after second transplantation) in the recipient spleen analyzed using flow cytometry. (**a**) Gating strategy, (**b**) fractions of B220+CD21/CD35loIgM+ transitional B cells, (**c**) B220+CD21/CD35hiIgM+ marginal B cells, (**d**) B220+ CD21/CD35+IgM+ follicular B cells, and (**e**) B220+CD138-CD38+IgM-IgD- memory B cells. Error bars represent 2 standard errors (SE).

Next, B cell fractions in the spleen were also observed by flow cytometry (Figure 3a–e). Fractions of transitional cells were significantly increased in the BAFF group compared to the other groups (1st TP CONT 15.8 ± 0.9 vs. Syngenic CONT 19.3 ± 2.1 vs. Allogenic CONT 16.2 ± 0.8 vs. BAFF group, 38.8 ± 15.6 vs. IgG1 CONT, 13.8 ± 1.1, *p* < 0.05, Figure 3b). Fractions of marginal cells (1st TP CONT 13.7 ± 0.9 vs. Syngenic CONT 12.2 ± 1.7 vs. Allogenic CONT 14.0 ± 1.9 vs. BAFF group, 6.4 ± 2.8 vs. IgG1 CONT, 10.5 ± 4.3, *p* < 0.05, Figure 3c) and follicular cells (1st TP CONT 67.3 ± 1.2 vs. Syngenic CONT 67.9 ± 0.85 vs. Allogenic CONT 69.8 ± 1.5 vs. BAFF group, 45.5 ± 7.8 vs. IgG1 CONT, 67.1 ± 3.6, *p* < 0.05, Figure 3d) however, were significantly decreased in the BAFF group. Memory cells were increased in the BAFF group compared to the other groups (1st TP CONT 0.3 ± 0.1 vs. Syngenic CONT 0.2 ± 0.1 vs. Allogenic CONT 0.3 ± 0.2 vs. BAFF group, 3.0 ± 3.4, *p* < 0.05; BAFF group vs. IgG1 CONT, 0.5 ± 0.2, *p* = 0.065, Figure 3e).

### 2.4. Comparison of T Cell Fractions in the Spleen

**Figure 4:**
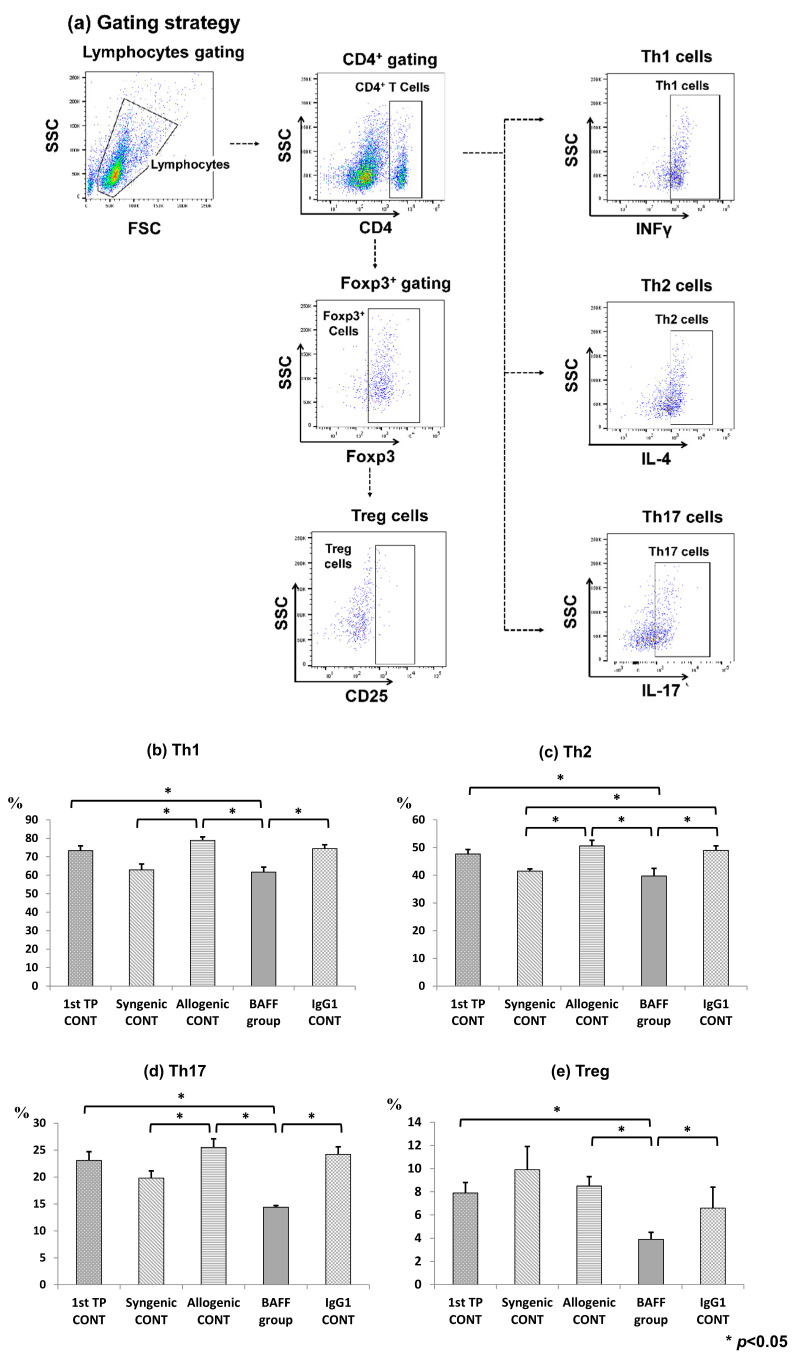
T cell population at week 9 (4 weeks after second transplantation) in the recipient spleen analyzed using flow cytometry. (**a**) Gating strategy and fractions of (**b**) CD4+/INFγ+ Th1 cells, (**c**) CD4+/IL4+ Th2 cells, (**d**) CD4+/IL-17+ Th17 cells, and (**e**) CD4+/CD25+/Foxp3+ Treg cells. Error bars represent 2 standard errors (SE).

BAFF inhibition mainly effects B cell maturation and activation but effects have also been observed in T cell activation. Therefore, T cell fractions in the spleen were also observed by flow cytometry (Figure 4a–e). Our results showed that Th1, Th2, Th17 and regulatory T cells (Treg) cells were all significantly inhibited in the spleen of the BAFF group compared to the IgG1 CONT group (Th1: 1st TP CONT 73.6 ± 3.4 vs. Syngenic CONT 62.9 ± 4.1 vs. Allogenic CONT 78.9 ± 2.5 vs. BAFF group 59.8 ± 4.1 vs. IgG1 CONT 74.5 + 2.7, *p* < 0.05) (Th2: 1st TP CONT 47.6 ± 1.9 vs. Syngenic CONT 41.9 ± 1.1 vs. Allogenic CONT 50.5 ± 2.7 vs. BAFF group 38.3 ± 4.3 vs. IgG1 CONT 49.0 + 2.1, *p* < 0.05) (Th17: 1st TP CONT 23.5 ± 2.0 vs. Syngenic CONT 19.7 ± 2.9 vs. Allogenic CONT 26.3 ± 2.1 vs. BAFF group 14.3 ± 0.4 vs. IgG1 CONT 24.2 + 1.9, *p* < 0.05) (Treg: 1st TP CONT 7.9 ± 1.2 vs. Syngenic CONT 9.7 ± 2.4 vs. Allogenic CONT 7.9 ± 1.1 vs. BAFF group 3.9 ± 0.9 vs. IgG1 CONT 7.2 + 2.5, *p* < 0.05).

### 2.5. Cell Surface BAFFR Expression on B Cells of the Bone Marrow and Spleen

**Figure 5:**
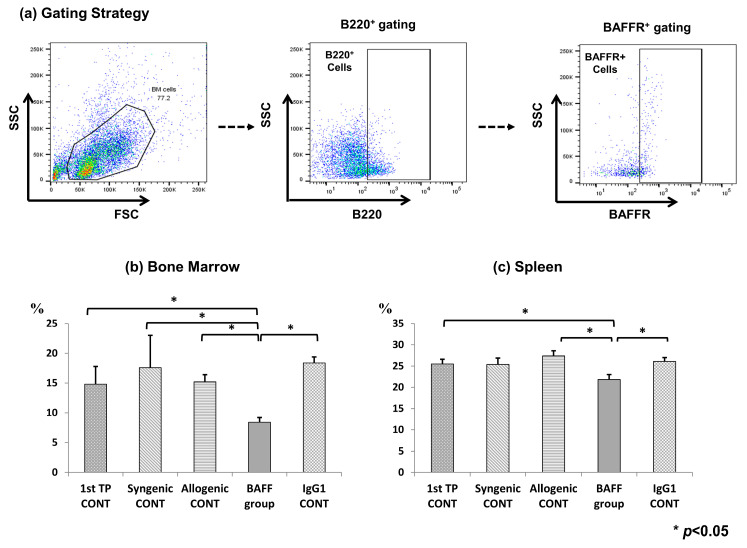
B cell activating factor receptor (BAFFR) expression at week 9 (4 weeks after second transplantation) in the recipient bone marrow and spleen cells analyzed using flow cytometry. (**a**) Gating strategy, (**b**) CD45R(B220)^+^/CD268(BAFFR)^+^) in bone marrow cells, (**c**) BAFFR (CD45R(B220)^+^/CD268(BAFFR)^+^) in spleen cells. Error bars represent 2 standard errors (SE).

We further observed BAFF receptor (BAFFR) expression on B cell surfaces in the bone marrow and spleen to determine whether BAFF inhibition effectively suppressed cell surface BAFFR expression (Figure 5a–c). BAFFR expression was significantly decreased in the BAFF group compared to the control groups in both the bone marrow (1st TP CONT 16.4 ± 4.5 vs. Syngenic CONT 17.9 ± 6.7 vs. Allogenic CONT 14.9 ± 3.5 vs. BAFF group, 8.6 ± 1.2 vs. IgG1 CONT, 17.8 ± 1.6, *p* < 0.05) and spleen (1st TP CONT 25.6 ± 1.7 vs. Syngenic CONT 25.2 ± 1.8 vs. Allogenic CONT 27.6 ± 1.8 vs. BAFF group, 21.4 ± 1.9 vs. IgG1 CONT, 26.6 ± 1.4, *p* < 0.05).

### 2.6. Microarray Analysis of the Sensitized Mouse and BAFF Inhibition Models

**Figure 6:**
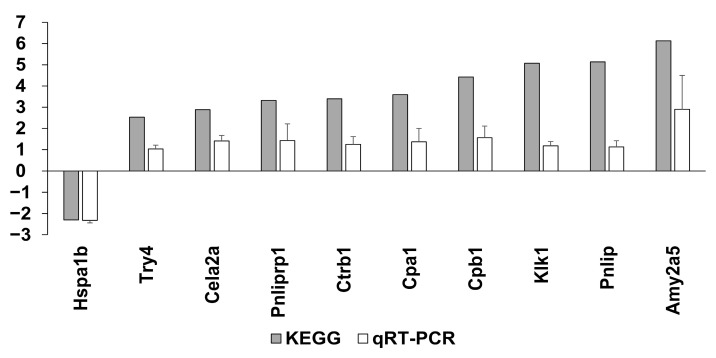
Validation of differentially expressed genes (DEGs) using qRT-PCR. Error bars represent 2 standard errors (SE).

To determine which gene probes are involved in BAFF inhibition, we compared the BAFF group and IgG1 CONT group, and 19 gene probes were found to be differently significantly regulated (seven genes were downregulated and 12 genes were upregulated in BAFF group compared to IgG1 CONT group) (Table 1, *p* < 0.05). Downregulated genes were associated with antigen binding, immune responses and immunoglobulin production, while upregulated genes were associated with antigen binding, immune responses and immunoglobulin production, as well as lipid binding CD4+ T cell control. We organized genes according to KEGG genes and 24 genes in total were significantly up- or down-regulated (Table S1, *p* < 0.05). Among them the heat shock protein (HSP) A1B was significantly upregulated in the IgG1 CONT group (fold change (FC) 1.85, *p* < 0.001), and suppressed in the BAFF group (FC-2.30, *p* < 0.001). Microarray results were validated using qRT-PCR (Figure 6). Ten candidate genes including Hspa1b, Try4, Cela2a, Pnlilprp1, Ctrb1, Cpa1, Cpb1, Klk1, Pnlip, and Amy2a5 were selected and analyzed. Although fold changes varied between the two methods, up- and down-regulation trends were consistent, therefore indicating that the microarray results were reliable.

### 2.7. GSEA Pathways Involved in BAFF Inhibition

**Figure 7:**
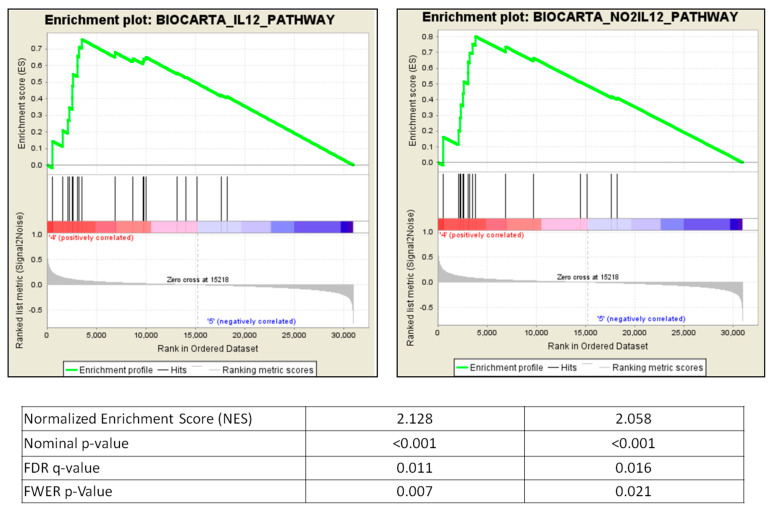
Top 2 most significantly enriched GSEA pathways in BAFF inhibition.

Based on the above results, differences of normalized mRNA expression between groups were ranked, and then tested using Gene Set Enrichment Analysis (GSEA). To observe pathways involved in BAFF inhibition, we compared the BAFF group and IgG1 CONT. In total, 23 gene sets were upregulated for BAFF inhibition, meeting significance (nominal *p*-value < 0.05, false discovery rate (FDR) < 25%, Table 2). The top two most significantly enriched gene sets involved in BAFF inhibition were the IL12 pathway (normalized enrichment score (NES) 2.128, nominal *p*-value < 0.001, FDR *q*-value 0.011) and NO2IL12 pathway (NES 2.058, nominal *p*-value < 0.001, FDR *q*-value 0.016) (Figure 7). Most significantly enriched transcripts in the BAFF group were CCR5, IL18R1, IL12A, and IL12RB2 (Table 3).

### 2.8. Changes of Immune Cell Fractions during Sensitization Using CIBERSORT

We further analyzed the microarray mRNA data using the CIBERSORT method to observe the changes of immune cell fractions during sensitization. For this, we compared the 1st TP group with the Syngenic CONT and Allogenic CONT groups. For the LM22 gene signature file (Table S2) only the macrophage subtypes showed significant differences between the three groups. M0 and M1 fractions were increased and M2 fractions decreased in 1st TP group compared to the Syngenic CONT and Allogenic CONT groups, but no significant differences were found between the Syngenic and Allogenic CONT groups. For the xCell gene signature file (Table S3), Th2 cell fractions were significantly low in the Allogenic CONT compared to the other two groups. These findings may be because inflammatory responses associated with the skin graft process itself overwhelmed immune responses associated with sensitization, and also because of the small number of mice in each group of our study.

## 3. Discussion

In this study, we found that BAFF inhibition effectively suppresses the development of anti-HLA-A2 Ab in the well-established highly sensitized mouse model. In addition, the suppression of anti-HLA-A2 Ab formation is accompanied by the regulation of B cell fractions in both the bone marrow and spleen, and also by changes of molecular signature in the transcript level. Our results suggest that BAFF inhibitors may be proposed as potential therapeutic agents for the prevention of allo-sensitization to HLA in patients waiting for transplantation.

First, we investigated the changes of anti-HLA-A2 Ab titers with or without BAFF inhibitor treatment. In regard to the sensitization model, we performed two skin grafts because, theoretically, memory B cells to HLA.A2 will be formed by the first exposure to a specific antigen, and a second exposure to the antigen is necessary for these memory B cells to expand and differentiate into antibody secreting plasma cells, which will ultimately produce the so-called “highly sensitized state” [19,20,21]. Indeed, the kinetics of DSA production observed in this model is consistent with previous descriptions of primary and secondary antibody responses. As we expected, anti-HLA.A2 Ab was not detected in the non-sensitized Syngenic CONT group during the study period (2 weeks to 7 weeks). However, in the allogenic transplant groups (Allogenic CONT, BAFF and IgG1 CONT), anti-HLA.A2 Ab titers rose in all groups after the first skin graft in a similar pattern, and in the allognenic CONT and IgG1 CONT, there were further increases after the second graft, which suggests that there were secondary antibody responses [21]. In contrast, antibody levels showed a dramatic decrease in the BAFF group, which demonstrates the suppressive effects of BAFF inhibition on anti-HLA.A2 Ab formation.

Second, to investigate the underlying cellular mechanisms for the inhibition of anti-HLA.A2 Ab formation, we analyzed immune cells belonging to the B cell lineage in the spleen and bone marrow using flow cytometry. As previously shown in studies with genetic BAFF ablation [22] or anti-BAFF blockade [23], BAFF inhibition spared pre-pro, immature and transitional B cells while depleting mature, marginal and follicular B cells. This is because the action of BAFF in the differentiation of immature and transitional B cells into mature, follicular and marginal zone B cells, and in the maintenance of these cells, has been suppressed. BAFFR expressed on B cell surfaces of both bone marrow and spleen cells were also effectively suppressed by BAFF inhibition. Meanwhile, there was a slight increase in memory B cells and LLPC cells. Murine memory B cells express transmembrane activator and CAML interactor (TACI) but not B cell maturation antigen (BCMA) or BAFFR, and the survival and function of memory B cells are BAFF and a proliferation inducing ligand (APRIL) independent. Human CD27+ memory B cells on the other hand, express high levels of BAFFR and TACI, with detectable levels of BCMA. Therefore, as shown in previous studies, BAFF inhibition in humans suppresses memory B cells in vivo [17] but in murine studies neutralization of both BAFF and APRIL did not affect memory B cells in vivo [24]. LLPCs express TACI and BCMA, therefore BAFF blockade alone does not impair LLPC survival. Blockade of both BAFF and APRIL is required for suppression of plasma cells in the bone marrow [7,25].

It may seem paradoxical that anti-HLA-A2 Ab showed significant decrease after BAFF inhibition even though memory B cells in the spleen or LLPC in the bone marrow did not decrease. One possible reason is that we did not measure IgG-specific B cells or plasma cells. Indeed, humans and mice with genetic BAFF- or BAFFR-deficiency in B cells have few circulating B cells, very low IgM and IgG concentrations but high IgA levels due to the development of IgA-secreting plasma cells in the gut [26]. In addition, BAFF inhibition has shown pro-regulatory effects on B cell compartments in murine and human studies. A recent human study using belimumab demonstrated an increase in B cells secreting the pro-regulatory IL-10 compared to IL-6 [17]. The effects of BAFF inhibition on the blockade of plasma cell survival niches in the bone marrow may affect the plasma cell pool with chronic inhibition [27], and BAFF blockade also prevents activation of dendritic cells in inflammatory tissues, resulting in a decrease in IL-6, maturation of Th17 cells and consequently a decrease in plasma cell survival [7,28]. This may contribute to the decreased production of HLA-A2 Ab after BAFF inhibition.

It was interesting to see in our study that all T cell fractions were suppressed. Many studies have already demonstrated the suppressive effects of BAFF inhibition on T helper cell activation and cytokine production [29]. Besides the direct suppression of stimulatory effects on T cell vitality, suppressive effects on B cells by BAFF inhibition result in secondary suppression of T cell expansion [30]. There have been some contradicting results on the effects of BAFF inhibition on the Treg compartment, as some have reported an expansion of this compartment [31,32], while we found this fraction to be suppressed as well. Stohl et al. [30] suggested that Treg cell expansion requires both BAFF stimulation and sufficient B cells; therefore, in pharmacologic neutralization of BAFF with sufficient reduction in B cells, one may expect a decrease in Treg cells. However, these results are divergent and further studies are needed to clarify this issue.

Lastly, we performed microarray analysis to investigate molecular signatures associated with the development or suppression of sensitization to HLA. One of our findings is that the HSPA1B was upregulated in sensitized mice and downregulated by the administration of BAFF mAb. The HSPA1B gene, also known as heat shock 70 kDa protein 1B encodes a 70 kDa heat shock protein (Hsp), a member of the heat shock protein 70 family, is known to be induced by ischemia, reperfusion and surgical stress. The role of heat shock proteins in transplantation is controversial. Hsps have previously been known to induce pro-inflammatory responses and have been associated with allograft rejection. For example, in rats, Hsp70 gene and protein expression were increased in rejected cardiac allografts [33], and in humans, Hsp 70 expression was found to be upregulated in deceased donor kidney transplants after post-ischemic reperfusion injury [34]. However, Hsps have also been proposed to be cytoprotective and have shown to improve organ viability after ischemia-perfusion injury in experimental models. Whether the suppression of Hsp70 by BAFF inhibition was due to attenuated immune responses against donor histocompatibility alloantigens or merely the result of a lesser injured allograft is yet to be known and will pose as an interesting topic to study in future experiments. In GSEA pathway analysis, IL12 and NO2IL12 pathways and CCR5, IL18R1, IL12A, and IL12RB2 transcripts were most significantly enhanced on GSEA of BAFF-inhibited groups. CCR5 has been known to be associated with the movement and maturation of progenitor B cells in the BM [35], while IL12 and 18 pathways were reported to be associated with the inhibition of B cell differentiation [36]. IL-12 is also known to induce Th1 cell differentiation and promote cell-mediated immunity and promote antibody-mediated immune responses via the differentiation of CD4+ T follicular helper cells [37]. IL-12 is known to have both pro- and anti-inflammatory potentials and further relationships between IL12 pathways and BAFF inhibition need to be elucidated.

There may be some concerns on how to apply the results of this study to the clinical setting. Indeed, some pilot trials using BAFF inhibitors alone for desensitization did not show meaningful results [16]. Previous trials using atacicept (an inhibitor of both BAFF and APRIL) in the treatment of lupus nephritis patients effectively suppressed all fractions of B cells including LLPC but resulted in severe hypogammaglobulinemia and severe infections [38]. BAFF inhibition suppresses B cell activation while keeping intact pathogen-induced immunological memory and pro-regulatory B cell function, and this may present as a therapeutical advantage. Additionally, as mentioned above, B cell depletion also results in impaired CD4+ T cell activation, therefore in-part supporting the effects of T cell inhibition [31]. Therefore, a combination therapy with a proteasome inhibitor to suppress LLPC, T cell inhibition using standard immunosuppressants, and suppression of germinal center reactivation and enhancing pro-regulatory B cell functions using BAFF blockade, may be the idealistic desensitization therapy. In this context, in a recent study, the use of BAFF inhibition in conjunction with standard maintenance immunosuppressive agents after kidney transplantation has shown promising results in suppressing de novo DSA formation and activation of B cells [17]. In addition, the results of a recent clinical trial using belimumab in combination with bortezomib for desensitization therapy in sensitized patients are still pending (NCT02500251).

One of the major limitations of our study is the small number of animals per group. We performed repeat experiments for internal validation and quality control. Another limitation is that we observed the effects of BAFF inhibition on B cell fractions and microarray molecular signatures but did not perform experiments to show the influences of BAFF inhibition on cellular functions or to clarify the relationship of microarray signatures and altered phenotypes in BAFF inhibition. Single cell RNA studies are being considered for future studies.

In conclusion, anti-BAFF monoclonal antibody inhibited pathways involved in B cell maturation, resulting in the reduction in HLA.A2-specific IgG and significantly inhibiting differentiation and maturation of B cells in both the bone marrow and spleen of the sensitized mouse model. Our data suggest that BAFF suppression may serve as a useful future strategy in combination with other agents for desensitization therapy.

## 4. Materials and Methods

### 4.1. Animals

Eight to twelve-week-old homozygous transgenic C57BL/6-Tg(HLA-A2.1)1Enge/J male mice and wild-type C57BL/6 mice weighing 25–30 g were purchased from the Jackson Laboratory (Jackson Laboratory, Bar Harbor, Maine, USA). All mice were housed in a specific pathogen-free facility in individual cages with temperature and light-controlled environments. All procedures were performed in accordance with the Laboratory Animals Welfare Act, the Guide for the Care and Use of Laboratory Animals (National Institute of Health publication no. 80-23, revised 1996) and were approved by the College of Medicine, Catholic University of Korea, Institutional Animal Care and Use Committee (CUMC 2017-00147-03).

### 4.2. Skin Allograft Transplant Procedure

Wild-type C57BL/6 mice were sensitized with skin allografts from C57BL/6-Tg(HLA-A2.1)1Enge/J mice according to murine skin graft models described previously [39]. Both donor and recipient strains share a common B6 genetic background except for a single transgenic HLA.A2 antigen. The expression of this antigen on donor cells, triggers alloimmune responses in the recipients resulting in production of HLA.A2-specific antibodies (anti-HLA.A2 Ab). Donor and recipients were anesthetized with intraperitoneal (i.p.) injections of Zoletil^®^50 (Tiletamine and Zolazepam) 30 mg/kg and Rompun^®^ (Xylazine) 10 mg/kg. Tail skin segmented to 8 × 8^−10^ mm^2^ sizes from C57BL/6-Tg(HLA-A2.1)1Enge/J mice was grafted onto the dorsal area of the C57BL/6 mice.

### 4.3. Experimental Design

**Figure 8:**
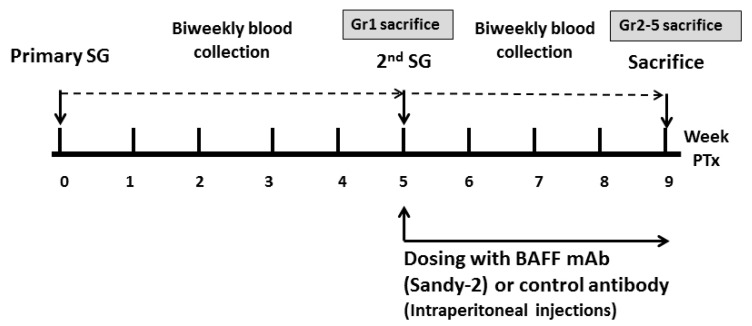
The Study Protocol. Abbreviations: SG, skin graft; Gr, group; PTx, post-transplant; BAFF, B cell activating factor; mAb, monoclonal antibody.

Mice were randomized into 5 groups (Group 1: 1st TP CONT, Group 2: Syngenic CONT Group 3: Allogenic CONT, Group 4: BAFF group, Group 5: IgG1 CONT) as shown in Table 4. There were 3 animals per group and experiments were repeated at least once for validation. Group 1 received just one skin transplant while the other 4 groups were re-immunized with a second skin allograft 5 weeks after the first transplant. Group 2 received syngenic transplants (from B6 to B6 mice) and Groups 3, 4, and 5 underwent 2 allogenic transplants. The BAFF group was treated with a single dose of anti-BAFF mAb (named Sandy-2, Adipogen^®^ Life Sciences, San Diego, CA, USA) at 2 mg/kg by i.p. injection an hour before the second skin graft transplantation. Group 5 was treated with the same dose of control IgG1 antibody i.p. an hour before the second skin graft transplantation (Figure 8). The dosing strategy was derived from a previous study in which Sandy-2 effectively blocked recombinant and endogenous BAFF in vitro and in vivo [40]. Mice were sacrificed using a CO2 chamber. Mice in the 1st TP CONT group were sacrificed at week 5, when the other 4 groups received the second skin transplant, and these were sacrificed 4 weeks after the second skin transplant. Mice spleen and bone marrow from femoral bone were harvested.

### 4.4. Measurement of Serum Donor-Specific Anti-HLA.A2 Antibodies

Blood samples were taken from the facial vein on weeks 0, 2, and 5 for all 5 groups just before each skin graft. For groups receiving a second skin graft, blood samples were additionally taken at week 7. Donor-specific anti-HLA A2 Ab was analyzed using LAB Screen Mixed assay (One Lambda, A Thermo Fisher Scientific Brand, Canoga Park, CA, USA) and LAB Screen Single Antigen (One Lambda) on a LAB Scan 3D system (One Lambda) according to the manufacturer’s specifications. Results were expressed as mean fluorescence intensity (MFI).

### 4.5. Flow Cytometry Analysis

Freshly isolated spleen cells were obtained by gently milling mice spleen in phosphate-buffered saline (PBS). Bone marrow cells were extracted from the femur bone of mice. Collected cells (1 × 106 cells/mL) were stained with anti B220-efluor 450 (clone; RA3-6B2, Thermo Fisher Scientific Inc., NY, USA), anti IgM-PE (clone; 11/41, Thermo Fisher Scientific Inc.), anti IgD-PerCPeflour 710 (clone; 11-26C, Thermo Fisher Scientific Inc.), anti CD21/CD35-APC (clone; 7G6, BD Biosciences, San Jose, CA, USA), anti CD38-FITC (clone:90, Thermo Fisher Scientific Inc.) and anti CD138-BV605 (clone; 281-2, BD Biosciences) monoclonal antibodies to observe the different B cell subsets (Table S4). For observation of T cell subsets, cells were stimulated for 4 h with phorbol 12-myristate 13-acetate (Sigma) and ionomycin (Sigma) with the addition of GolgiStop (BD Bioscience). Stimulated cells were stained with the following antibodies: anti CD4-FITC (clone:RM4-5), anti CD25-eFluor 450 (clone:PC61.5), anti IL-17-PE (clone:eBio17B7), anti Foxp3-APC (clone:FJK-16S), anti IFNγ-APC (clone:XMG1.2), and anti IL-4-PE-Cy 7 (clone:BVD6-24G2, all from eBioscience, San Diego, CA, USA). Intracellular staining was performed using an intracellular staining kit (BD Biosciences or eBioscience) according to the manufacturer’s protocol. To observe cell-surface BAFFR concentrations, cells were stained with anti-CD45R (B220, Millipore-Sigma) and anti-CD268(BAFFR, BD Biosciences). Flow cytometric analysis was performed using a fluorescence-activated cell sorting (FACS) Fortessa instrument (BD Biosciences, San Jose, CA, USA) and data were analyzed using Flow Jo Version 10.0.6 software (Tree Star, Ashland, OR, USA).

### 4.6. Microarray Analysis

#### 4.6.1. mRNA Extraction and Quality Control

Following the manufacturer’s instructions, mRNA was extracted from mice spleen cells using the Relia Prep RNA Miniprep Systems (Promega Corporation, Madison, WI, USA). RNA purity and integrity were assessed using Agilent 2100 Bioanalyzer (Agilent Technologies, Palo Alto, CA, USA) and the optical density (OD) 260/280 ratio was calculated to indicate nucleic acid purity.

#### 4.6.2. Affymetrix Whole Transcript Expression Arrays Methods

The Affymetrix Whole transcript Expression array process was performed as recommend by the manufacturer (GeneChip Whole Transcript PLUS reagent Kit, Thermo Fisher Scientific, Waltham, MA, USA), and cDNA was synthesized using the GeneChip WT (Whole Transcript) Amplification kit as previously described [41]. Approximately 5.5 μg of fragmented sense cDNA was biotin-labeled with TdT (terminal deoxynucleotidyl transferase) using the GeneChip WT Terminal labeling kit and hybridized to the Affymetrix GeneChip Mouse 2.0 ST Array at 45 °C for 16 h. Hybridized arrays were washed and stained on a GeneChip Fluidics Station 450 and scanned on a GCS3000 Scanner (Affymetrix, Santa Clara, CA, USA). Signal values were computed using the Affymetrix^®^ GeneChip™ Command Console software.

#### 4.6.3. Raw Data Preparation and Analysis

Data were summarized and normalized by applying the robust multi-average (RMA) method and differentially expressed gene (DEG) analysis. Gene-Enrichment and Functional Annotation analysis for determining significant probes was performed using Gene Ontology (www.geneontology.org/) and KEGG (www.genome.jp/kegg/) [42]. Differences of normalized mRNA expression between groups were also ranked, and then tested using Gene Set Enrichment Analysis (GSEA) using curated gene sets (KEGG, BIOCARTA, REACTOME and GO) [43]. DEGs were validated using qRT-PCR.

#### 4.6.4. Validation Using Quantitative Real-Time PCR (qRT-PCR)

To validate the microarray results, 10 candidate genes were selected and analyzed using qRT-PCR. Using a Dyne 1st-Strand cDNA Synthesis Kit (Dyne Bio Inc., Seung-Nam, Korea), five micrograms of purified RNA were reverse transcribed into first-strand complementary DNA. RT-q PCR amplification was performed using the SYBR Green Premix in the Light Cycler 480 system (Roche, Rotkreuz, Switzerland). The relative mRNA expression levels were normalized to the GADPH gene using the change in cycle threshold method. The primer sequences used for qPCR are listed in Table S5.

#### 4.6.5. Subanalysis of Microarray Results Using CIBERSORT

Microarray mRNA data were further analyzed with CIBERSORT, which is an analytical tool that can be used to characterize immune cell composition in a complex tissue by quantifying the gene expression of different immune cell types (http://cibersort.stanford.edu) [44]. We applied the LM22 gene signature file, which consists of 22 immune cell subsets, and xCell, which consists of 60 immune cell and stromal signatures.

### 4.7. Statistical Analysis

Results are shown as mean ± standard deviation (SD) or median ± interquartile range (IQR) and comparisons were made with student’s *t*-test or one-way analysis of variance for parametric data and Mann–Whitney U test or Kruskal–Wallis test for non-parametric data. Statistical analysis was performed using SPSS version 20 for Windows (SPSS Inc., Chicago, IL, USA). *p* < 0.05 was considered statistically significant. Statistical significance of the microarray expression data was determined using the local-pooled-error (LPE) test and fold change in which the null hypothesis was that no difference exists among groups. FDR was controlled by adjusting the *p*-value using the Benjamini–Hochberg algorithm. Hierarchical cluster analyses of the DEG sets were performed using complete linkage and Euclidean distance as a measure of similarity. All data analysis and visualization of differentially expressed genes was conducted using R 3.3.3 (www.r-project.org). A nominal *p*-value < 0.05 and FDR < 25% was considered significant.

## Figures and Tables

**Table 1:** Significantly different gene probes between second allogenic TP and BAFF inhibitor group (BAFF group) and second allogenic TP and control IgG1 (IgG1 CONT) group.

Symbol	Gene Annotation	Function	FC	*p*
Ighv1-78	immunoglobulin heavy variable 1-78	antigen binding and immunoglobulin production	−3.023	0.000
Ighv1-31	immunoglobulin heavy variable 1-31	antigen binding and immunoglobulin production	−2.779	0.011
Igkv3-4	immunoglobulin kappa variable 3-4	Immune response and immunoglobulin production	−2.531	0.001
Gm24762	predicted gene, 24762		−2.116	0.011
Igkv9-123	immunoglobulin kappa variable 9-123	Immune response and immunoglobulin production	−1.974	0.042
Igkv4-55	immunoglobulin kappa variable 4-55	antigen binding and immunoglobulin production	−1.614	0.005
Ighv1-7	immunoglobulin heavy variable V1-7	antigen binding and immunoglobulin production	−1.610	0.011
Apol11b	apolipoprotein L 11b	Lipid binding	1.733	0.001
Igkv11-125	immunoglobulin kappa variable 11-125	Immune response and immunoglobulin production	2.027	0.006
Ighv1-39	immunoglobulin heavy variable 1-39	antigen binding and immunoglobulin production	2.074	0.025
Ighv9-1	immunoglobulin heavy variable 9-1	antigen binding and immunoglobulin production	2.242	0.000
Igkv1-122	immunoglobulin kappa chain variable 1-122	Immune response and immunoglobulin production	2.265	0.000
Igkv14-126	immunoglobulin kappa variable 14-126	Immune response and immunoglobulin production	2.436	0.000
Mir669a-1	microRNA 669a-1	negative regulation of skeletal muscle cell differentiation and regulation of gene expression	2.526	0.011
Mir669p-1	microRNA 669p-1	Regulatory, pathogenic and control CD4+ T cells	2.572	0.011
Ear1	eosinophil-associated, ribonuclease A family, member 1	Endonuclease activity, hydrolase activity, nuclease activity, ribonuclease activity	2.599	0.002
Ighv12-3	immunoglobulin heavy variable V12-3	antigen binding and immunoglobulin production	2.812	0.000
Apol11a	apolipoprotein L 11a	Lipid binding	3.039	0.000
Ighv1-58	immunoglobulin heavy variable 1-58	antigen binding and immunoglobulin production	3.739	0.011

**Table 2:** Most significantly enriched Gene Set Enrichment Analysis (GSEA) pathways in BAFF group vs. IgG1 CONT.

Pathways	Size	ES	NES	NOM *p*-Value	FDR *q*-Value	FWER *p*-Value
BIOCARTAIL12PATHWAY	21	0.76	2.13	0	0.011	0.007
BIOCARTANO2IL12PATHWAY	17	0.8	2.06	0	0.017	0.021
BIOCARTACSKPATHWAY	19	0.75	2.03	0	0.014	0.027
PIDIL12STAT4PATHWAY	31	0.65	1.96	0.002	0.047	0.111
PIDIL122PATHWAY	57	0.56	1.94	0	0.047	0.138
BIOCARTANKCELLSPATHWAY	18	0.72	1.94	0	0.041	0.145
REACTOMEGENERATIONOFSECONDMESSENGERMOLECULES	20	0.72	1.93	0.002	0.042	0.171
BIOCARTACTLA4PATHWAY	16	0.73	1.92	0.006	0.039	0.18
KEGGNATURALKILLERCELLMEDIATEDCYTOTOXICITY	102	0.51	1.92	0	0.036	0.187
REACTOMETCRSIGNALING	44	0.57	1.86	0	0.078	0.401
REACTOMEDEGRADATIONOFTHEEXTRACELLULARMATRIX	25	0.63	1.85	0.007	0.082	0.449
KEGGADHERENSJUNCTION	73	0.52	1.84	0	0.078	0.459
PIDTCRPATHWAY	64	0.52	1.82	0.002	0.094	0.539
KEGGCYTOKINECYTOKINERECEPTORINTERACTION	224	0.43	1.82	0	0.089	0.546
BIOCARTATH1TH2PATHWAY	16	0.69	1.79	0.006	0.111	0.655
STTCELLSIGNALTRANSDUCTION	45	0.54	1.78	0.002	0.124	0.726
PIDPTP1BPATHWAY	49	0.54	1.75	0	0.168	0.84
REACTOMECOSTIMULATIONBYTHECD28FAMILY	54	0.5	1.75	0	0.16	0.842
PIDTCPTPPATHWAY	42	0.53	1.72	0	0.186	0.897
REACTOMEIMMUNOREGULATORYINTERACTIONSBETWEENALYMPHOIDANDANON-LYMPHOIDCELL	41	0.54	1.72	0.005	0.188	0.91
PIDINTEGRINA9B1PATHWAY	25	0.59	1.69	0	0.233	0.948
KEGGLYSOSOME	116	0.44	1.68	0.002	0.245	0.959

**Table 3:** Most significantly enriched transcripts in GSEA pathways in BAFF group vs. IgG1 CONT.

IL12 Pathway		NO2-il12 Pathway		CSK Pathway	
Probe	Rank Metric Score	Probe	Rank Metric Score	Probe	Rank Metric Score
CCR5	0.251	CCR5	0.251	CD4	0.119
IL18R1	0.151	IL12A	0.127	LCK	0.118
IL12A	0.127	IL12RB2	0.121	ZAP70	0.117
IL12RB2	0.121	CD4	0.119	CD3E	0.109
IL12RB1	0.111	IL12RB1	0.111	CD3D	0.097
ETV5	0.110	CD3E	0.109	CD3G	0.097
CD3E	0.109	CD3D	0.097	CD247	0.093
CD3D	0.097	CD3G	0.097		
CD3G	0.097	CD247	0.093		
CD247	0.093	CXCR3	0.087		
CXCR3	0.087	NOS2	0.082		

**Table 4:** Definition of experimental group.

Group	Name	Description
1	1st Allogenic TP (1st TP CONT)	HLA-A2→B/6
2	2nd Syngenic TP (Syngenic CONT)	B/6→B/6 × 2 times
3	2nd Allogenic TP (Allogenic CONT)	HLA-A2→B/6 × 2 times
4	2nd Allogenic TP + BAFF inhibitor (BAFF group)	HLA-A2→B/6 × 2 times Anti-BAFF mAb administration
5	2nd Allogenic TP + Control IgG1 (IgG1 CONT)	HLA-A2→B/6 × 2 times Control IgG1 Ab administration

## Data Availability

The data presented in this study are available on request from the corresponding authors.

## Supplementary Materials

The following are available online at https://www.mdpi.com/1422-0067/22/2/861/s1.

## Abbreviations

ABMR	Antibody-mediated rejection
APRIL BAFF	A proliferation inducing ligand B cell activating factor
BAFFR	B cell activating factor receptor
BCMA	B cell maturation antigen
DEG	Differentially expressed gene
DSA	Donor-specific antibody
FACS	Fluorescence-activated cell sorting
FDR	False discovery rate
FC	Fold change
GAPDH	Glyceraldehyde 3-phosphate dehydrogenase
GSEA	Gene Set Enrichment Analysis
Hsp	Heat shock protein
Anti-HLA.A2 Ab	HLA.A2-specific antibodies
HLA	Human leukocyte antibody
IQR	Interquartile range
IVIg	Intravenous immune globulin
KT	Kidney transplant
LPE	Local-pooled-error
LLPC	Long lived plasma cells
MFI	Mean fluorescence intensity
mAb	Monoclonal antibody
NES	Normalized enrichment score
OD	Optical density
PBS	Phosphate-buffered saline
qRT-PCR	Quantitative real-time polymerase chain reaction
RMA	Robust multi-average
SLE	Systemic lupus erythematosus
SD	Standard deviation
TdT	Terminal deoxynucleotidyl transferase
TACI	Transmembrane activator and CAML interactor
