# Supplementary material for: BAFF Inhibition Effectively Suppresses the Development of Anti-HLA.A2 Antibody in the Highly Sensitized Mouse Model
Source: Int J Mol Sci. 2021 Jan 16;22(2):861. doi: 10.3390/ijms22020861 (PMC7830620; doi:10.3390/ijms22020861)
Supplement: Supplementary file 1 [file ijms-22-00861-s001.pdf]

Table S1. Significantly up and down regulated KEGG genes between groups

Genes	Syngenic vs. allogenic CONT		BAFF group vs. IgG1 CONT	
	Fold change	P -Value	Fold change	P-Value
2210010C04Rik	-4.480594	0.000	2.863163	0.000
Amy2a5	-7.459609	0.004	6.124562	0.032
Amy2b	-1.542093	0.000		
Cel	-1.542093	0.000	3.110601	0.000
Cela2a	-4.327149	0.000	2.880249	0.000
Cela3b	-3.598960	0.000	2.179056	0.000
Clps	-2.529198	0.000	2.711172	0.000
Cpa1	-3.423044	0.000	3.593011	0.000
Cpa2	-2.675633	0.000	2.201917	0.000
Cpb1	-5.171227	0.000	4.422953	0.000
Ctrb1	-4.469789	0.000	3.397052	0.000
Ctrl	-3.851606	0.000	2.530273	0.000
Hspa1a	1.639978	0.000		
Hspa1b	1.853801	0.000	-2.300823	0.000
Pla2g1b	-3.660354	0.004		
Klk1	-4.515691	0.040	5.074926	0.005
Klk1b5	-2.174193	0.040		
Mir103-2	-1.771699	0.041		
Mir145a	1.505348	0.041		
Mirlet7f-1	-2.083083	0.041		
Pla2g1b	-3.660354	0.004		

Pnlip	-7.559042	0.000	5.140612	0.000
Pnliprp1	-3.649242	0.003	3.319709	0.000
Prss2	-2.266930	0.000		
Try4	-2.614992	0.000	2.532192	0.000

Abbreviations: KEGG, Kyoto Encyclopedia of Genes and Genomes; BAFF, B cell activating factor

Table S2. Immune cell fractions using CIBERSORT, LM22 gene signature file

Immune cell type	Mean ± SD			P-value		
	1st TP CONT	Syngenic CONT	Allogenic CONT	1st TP vs. Allogenic	1st TP vs. Syngenic	Syngenic vs. Allogenic
B cells naïve	0.413 ± 0.036	0.375 ± 0.039	0.361 ± 0.017	0.127	0.127	0.827
B cells memory	0.072 ± 0.034	0.085 ± 0.036	0.093 ± 0.021	0.275	0.827	0.827
Plasma cells	0.077 ± 0.014	0.106 ± 0.006	0.098 ± 0.009	0.127	0.050	0.275
T cells CD8	0.086 ± 0.029	0.106 ± 0.006	0.114 ± 0.019	0.436	0.793	1.000
T cells CD4 memory resting	0.069 ± 0.009	0.063 ± 0.019	0.056 ± 0.022	0.483	0.483	1.000
T cells CD4 memory activated	0.026 ± 0.014	0.010 ± 0.005	0.008 ± 0.006	0.102	0.145	0.958
T cells follicular helper	0.000 ± 0.000	0.006 ± 0.011	0.008 ± 0.007	0.713	1.000	1.000
T cells regulatory (Tregs)	0.019 ± 0.007	0.023 ± 0.016	0.016 ± 0.009	0.933	0.935	0.768
NK cells resting	0.042 ± 0.018	0.021 ± 0.007	0.022 ± 0.008	0.193	0.163	0.990
NK cells activated	0.000 ± 0.000	0.001 ± 0.001	0.000 ± 0.000	0.975	0.493	0.609
Monocytes	0.120 ± 0.017	0.162 ± 0.019	0.140 ± 0.014	0.373	0.055	0.341
Macrophages M0	0.018 ± 0.006	0.003 ± 0.002	0.001 ± 0.001	0.004	0.006	0.855
Macrophages M1	0.036 ± 0.003	0.012 ± 0.002	0.007 ± 0.002	0.000	0.000	0.121
Macrophages M2	0.005 ± 0.004	0.047 ± 0.014	0.057 ± 0.008	0.001	0.004	0.464

Abbreviations: SD, standard deviation

Table S3. Immune cell fractions using CIBERSORT, xCell gene signature file

**Immune cell type**	**Mean ± SD**			**P-value**		
	**1st TP CONT**	**Syngenic CONT**	**Allogenic CONT**	**1st TP vs. Allogenic**	**1st TP vs. Syngenic**	**Syngenic vs. Allogenic**
B cells	0.008 ± 0.009	0.035 ± 0.009	0.026 ± 0.015	0.022	0.158	0.389
Class-switched memory B-cells	0.005 ± 0.007	0.041 ± 0.022	0.027 ± 0.006	0.050	0.014	0.374
Plasma cells	0.015 ± 0.013	0.043 ± 0.008	0.050 ± 0.015	0.034	0.040	0.509
Pro B cells	0.028 ± 0.012	0.030 ± 0.012	0.000 ± 0.000	0.507	0.093	0.423
CD4 T cells	0.011± 0.011	0.035 ± 0.031	0.025 ± 0.013	0.285	0.233	0.646
CD4 Tcm	0.004 ± 0.006	0.025 ± 0.022	0.021 ± 0.010	0.228	0.067	0.770
CD4 Tem	0.015± 0.017	0.038 ± 0.026	0.049 ± 0.009	0.278	0.038	0.876
CD4+ memory T-cells	0.043 ± 0.011	0.028 ± 0.021	0.031 ± 0.027	0.336	0.525	0.723
CD4+ naive T-cells	0.000 ± 0.000	0.018 ± 0.016	0.013 ± 0.016	0.199	0.305	0.374
CD8+ Tcm	0.002± 0.000	0.003± 0.000	0.016 ± 0.012	0.975	0.118	0.746
CD8 naïve T cell	0.004 ± 0.007	0.025 ± 0.002	0.013 ± 0.020	0.006	0.493	0.337
Tgd cells	0.053 ± 0.005	0.020 ± 0.034	0.003 ± 0.006	0.237	0.000	0.500
Th1	0.005 ± 0.008	0.033 ± 0.030	0.008 ± 0.014	0.191	0.764	0.259
Th2	0.034 ± 0.009	0.000 ± 0.001	0.009 ± 0.004	0.003	0.012	0.021
Tregs	0.002± 0.002	0.013 ± 0.012	0.011 ± 0.018	0.186	0.452	0.850
Epithelial cells	0.002 ± 0.001	0.025 ± 0.005	0.019 ± 0.002	0.002	0.000	0.143
Erythrocytes	0.150 ± 0.056	0.090 ± 0.091	0.030 ± 0.033	0.380	0.117	0.371

GMP	0.004± 0.012	0.012 ± 0.013	0.009 ± 0.013	0.071	0.049	0.795
HSC	0.048 ± 0.074	0.013 ± 0.015	0.026 ± 0.044	0.498	0.685	0.638
Keratinocytes	0.002 ± 0.002	0.005 ± 0.006	0.003 ± 0.001	0.411	0.540	0.545
MPP	0.057 ± 0.046	0.008 ± 0.014	0.012 ± 0.013	0.148	0.175	0.730
MSC	0.036 ± 0.037	0.057 ± 0.014	0.016 ± 0.027	0.407	0.477	0.077
Macrophages M1	0.009± 0.010	0.003± 0.004	0.021 ± 0.012	0.422	0.248	0.075
Macrophages M2	0.001± 0.002	0.001 ± 0.001	0.000 ± 0.000	0.761	0.423	0.423
Mast cells	0.002 ± 0.002	0.003 ± 0.003	0.004 ± 0.004	0.421	0.342	0.727
Megakaryocytes	0.017 ± 0.015	0.000 ± 0.000	0.033 ± 0.016	0.135	0.258	0.021
Melanocytes	0.007± 0.006	0.007 ± 0.002	0.006 ± 0.006	0.939	0.822	0.706
Mesangial cells	0.006 ± 0.003	0.003 ± 0.005	0.004 ± 0.007	0.414	0.641	0.861
Myocytes	0.002 ± 0.003	0.001 ± 0.001	0.009 ± 0.002	0.774	0.042	0.006
NK cells	0.037 ± 0.017	0.018 ± 0.015	0.014 ± 0.014	0.214	0.137	0.755
NKT	0.018 ± 0.018	0.053 ± 0.002	0.033 ± 0.019	0.031	0.371	0.142
Neurons	0.001 ± 0.001	0.001 ± 0.002	0.002 ± 0.001	0.745	0.485	0.765
Neutrophils	0.011 ± 0.019	0.009 ± 0.008	0.024 ± 0.028	0.831	0.563	0.426
Osteoblast	0.000 ± 0.000	0.003 ± 0.005	0.012 ± 0.021	0.423	0.413	0.514
Pericytes	0.003 ± 0.003	0.003 ± 0.003	0.001 ± 0.002	0.890	0.450	0.533
Platelets	0.036± 0.016	0.000 ± 0.000	0.024 ± 0.006	0.019	0.309	0.022
Preadipocytes	0.011 ± 0.006	0.003 ± 0.006	0.001 ± 0.002	0.174	0.053	0.615
Sebocytes	0.002 ± 0.001	0.002 ± 0.001	0.001 ± 0.001	0.958	0.615	0.639
Skeletal Muscle cells	0.008± 0.004	0.003± 0.004	0.000 ± 0.001	0.219	0.068	0.228

aDC	0.000 ± 0.000	0.002 ± 0.002	0.002 ± 0.004	0.184	0.374	0.864
cDC	0.001 ± 0.002	0.004 ± 0.005	0.000 ± 0.000	0.480	0.374	0.345
iDC	0.028 ± 0.012	0.030 ± 0.012	0.011 ± 0.009	0.885	0.115	0.092
lyEndothelial	0.009 ± 0.011	0.004 ± 0.007	0.019 ± 0.007	0.571	0.255	0.067
mvEndothelial	0.008 ± 0.007	0.010 ± 0.009	0.022 ± 0.006	0.751	0.056	0.113
pDC	0.001 ± 0.002	0.004 ± 0.005	0.005 ± 0.005	0.140	0.122	0.888
Immune Score	0.064 ± 0.030	0.082 ± 0.056	0.085 ± 0.036	0.659	0.487	0.937
Stroma Score	0.001 ± 0.002	0.003 ± 0.006	0.011 ± 0.002	0.614	0.004	0.100
Microenvironment Score	0.070 ± 0.032	0.085 ± 0.059	0.096 ± 0.036	0.641	0.336	0.799

Abbreviations: SD, standard deviation

Table S4. Fluorescence-activated cell sorting (FACS) markers

B cell subset	Surface marker
Pre-Pro B cell (BM)	B220+CD21/CD35-IgM-
Immature B cell (BM)	B220+CD21/CD35-IgM+
Mature/Recirculation (BM)	B220+ CD21/CD35+IgM+
Transitional B cell (SP)	B220+CD21/CD35loIgM+
Marginal zone B cell (SP)	B220+ CD21/CD35hiIgM+
Follicular B cell (SP)	B220+ CD21/CD35+IgM+
Long-lived Plasma cell (BM)	B220lowCD138+CD38low (Ig–)
Memory B cell (SP)	B220+CD138-CD38+IgM-IgD-

Abbreviations: BM, bone marrow; SP, spleen

Table S5. qRT-PCR validation of selected genes from the microarray analysis

Genes	Primer sequence (5′-3′) Forward	Primer sequence (5′-3′) Reverse
Hspa1b	TGGTGCAGTCCGACATGAAG	AGGTCGGTGTGAACGGATTTG
Try4	ATGAGAGCACTCCTGTTCCT	TTAGTTTGCAGCAATGGTGT
Cela2a	TGATGTGAGCAGGGTAGTTGG	CACTCGGTAGGTCTGATAGTTG
Pnliprp1	ATCAACACTCGCTTCCTGCT	TCAACCACCCAGTTCTCCTC
Ctrb1	GCAAGACCAAATACAATGCCC	TGCGCAGATCATCACATCG
Cpa1	GACGAGGAGAAGCAGCAGAT	GATGCCAGTGTCAATCCAGA
Cpb1	GGTTTCCACGCAAGAGAG	GTTGACCACAGGCAGAACA
Klk1	CCGCTTCACCAAATATCAATGTG	GCTCATCTGGGTATTCATATTTGACG
Pnlip	AGCCATTGGAAGGATCACAG	CGTCGATGTCAACAATTTGG
Amy2a5	TGCTTTCCCTCATTGGGTTC	AATATCAACCCAGCGCCACT

Abbreviations: qRT-PCR, Quantitative real-time polymerase chain reaction
